# Supplementary material for: Saudi radiology trainees’ insights on safety and professionalism in the workplace
Source: PeerJ. 2025 Apr 3;13:e19257. doi: 10.7717/peerj.19257 (PMC11972563; doi:10.7717/peerj.19257)
Supplement: Supplemental Information 2 [file peerj-13-19257-s002.pdf]

Questionnaire “Saudi Radiology Trainees’ Perceptions of Speaking Up Culture Related to Safty and Unprofessional Behavior in Their Work Environments”

King Abdullah International Medical Research Center
KAIMRC
APPROVED
IRB
Institutional Review Board

The questionnaire consists of two parts:

A) Personal information

B) Questions about your perceptions of the cultures regarding speaking up about safety concerns versus unprofessional behavior (consists of 5 sections each section consists of 2 questions)

C) Predictors Affecting the odds of speaking up about a medical error in the hypothetical vignette

A) Demofraphic information:

Please state your gender:

- ◯ Male
- ◯ Female

Please state your age:

- ◯ ≥ 30
- ◯ < 30

Your clinical experience:

- ◯ ≥ 5
- ◯ < 5

From which city you are:

- ◯ Jeddah
- ◯ Riyadh
- ◯ Al-Ahsa

B) Questions about your perceptions of the Cultures Regarding Speaking up About Safety Concerns Versus Unprofessional Behavior

Section 1: Colleague encouragement

Q1) I am encouraged by my colleagues to speak up about traditional patient safety concerns

- ◯ Strongly disagree
- ◯ Disagree
- ◯ Neutral
- ◯ Agree
- ◯ Strongly agree

King Abdullah International Medical Research Center
KAIMRC
APPROVED
IRB
Institutional Review Board

Q2) I am encouraged by my colleagues to speak up about unprofessional behavior

- ◯ Strongly disagree
- ◯ Disagree
- ◯ Neutral
- ◯ Agree
- ◯ Strongly agree

Section 2: Difficulty speaking up

Q1) In my clinical area, it is difficult to speak up if I have traditional patient safety concerns

- ◯ Strongly disagree
- ◯ Disagree
- ◯ Neutral
- ◯ Agree
- ◯ Strongly agree

Q2) In my clinical area, it is difficult to speak up if I observe unprofessional behavior

- ◯ Strongly disagree
- ◯ Disagree
- ◯ Neutral
- ◯ Agree
- ◯ Strongly agree

Section 3: Meaningful change

Q1) Speaking up about traditional patient safety concerns results in meaningful change in my clinical area

- ◯ Strongly disagree
- ◯ Disagree
- ◯ Neutral
- ◯ Agree
- ◯ Strongly agree

Q2) Speaking up about unprofessional behavior results in meaningful change in my clinical area

- ◯ Strongly disagree
- ◯ Disagree
- ◯ Neutral
- ◯ Agree
- ◯ Strongly agree

Section 4: Clinical culture

Q1) The culture in my clinical area makes it easy to speak up about traditional patient safety concerns that do not involve me or my patients

- ◯ Strongly disagree
- ◯ Disagree
- ◯ Neutral
- ◯ Agree
- ◯ Strongly agree

King Abdullah International Medical Research Center
KAIMRC
APPROVED
IRB
Institutional Review Board

Q2) The culture in my clinical area makes it easy to speak up about unprofessional behavior that does not involve me or my patients

- ◯ Strongly disagree
- ◯ Disagree
- ◯ Neutral
- ◯ Agree
- ◯ Strongly agree

Section 5: Observe others speaking up

Q1) In my clinical area, I observe others speaking up about traditional patient safety concerns even if they are not directly involved in the patient's care

- ◯ Strongly disagree
- ◯ Disagree
- ◯ Neutral
- ◯ Agree
- ◯ Strongly agree

Q2) In my clinical area, I observe others speaking up about unprofessional behavior even if they are not directly involved in the patient's care

- ◯ Strongly disagree
- ◯ Disagree
- ◯ Neutral
- ◯ Agree
- ◯ Strongly agree

C) Predictors Affecting the Odds of Speaking up About a Medical Error in the Hypothetical Vignette

Please review the following hypothetical scenario and answer the follow questions. Please respond to each case as though you were in your usual role.

You are working in the radiology suite when a clinician comes in to place a central catheter on the patient under radiographic guidance. The clini- cian sets up the supplies and prepares the patient. The clinician puts on a sterile gown and gloves. The clinician then accidentally places a gloved hand on a nonsterile part of the ultrasound machine and proceeds to grab the catheter, preparing to place the line.

Q1) How inclined would you be to speak up about the breach in sterile technique if the clinician was a(n): **(1–5, not at all likely–completely likely)**

King Abdullah International Medical Research Center
KAIMRC
APPROVED
IRB
Institutional Review Board

a. Nurse

- ◯ Not at all likely
- ◯ Unlikely
- ◯ Neutral
- ◯ Likely
- ◯ Completely likely

b. Intern

- ◯ Not at all likely
- ◯ Unlikely
- ◯ Neutral
- ◯ Likely
- ◯ Completely likely

c. Resident

- ◯ Not at all likely
- ◯ Unlikely
- ◯ Neutral
- ◯ Likely
- ◯ Completely likely

d. Attending

- ◯ Not at all likely
- ◯ Unlikely
- ◯ Neutral
- ◯ Likely
- ◯ Completely likely

Q2) What is the potential for harm to the patient in this situation? **(1–5, very low–very high)**

- ◯ Very Low
- ◯ Low
- ◯ Neutral
- ◯ High
- ◯ Very High
